# Comprehensive Analysis of Cancer-Proteogenome to Identify Biomarkers for the Early Diagnosis and Prognosis of Cancer

**DOI:** 10.3390/proteomes5040028

**Published:** 2017-10-25

**Authors:** Hem D. Shukla

**Affiliations:** 1 Department of Pharmaceutical Sciences, University of Maryland, Baltimore, MD 21201, USA; hshukla@ndm.edu; Tel.: +1-410-852-4502; 2 Department of Biology, Notre Dame of Maryland University, Baltimore, MD 21210, USA

**Keywords:** cancer proteome, biomarker, early diagnosis, prognosis, personalized medicine

## Abstract

During the past century, our understanding of cancer diagnosis and treatment has been based on a monogenic approach, and as a consequence our knowledge of the clinical genetic underpinnings of cancer is incomplete. Since the completion of the human genome in 2003, it has steered us into therapeutic target discovery, enabling us to mine the genome using cutting edge proteogenomics tools. A number of novel and promising cancer targets have emerged from the genome project for diagnostics, therapeutics, and prognostic markers, which are being used to monitor response to cancer treatment. The heterogeneous nature of cancer has hindered progress in understanding the underlying mechanisms that lead to abnormal cellular growth. Since, the start of The Cancer Genome Atlas (TCGA), and the International Genome consortium projects, there has been tremendous progress in genome sequencing and immense numbers of cancer genomes have been completed, and this approach has transformed our understanding of the diagnosis and treatment of different types of cancers. By employing Genomics and proteomics technologies, an immense amount of genomic data is being generated on clinical tumors, which has transformed the cancer landscape and has the potential to transform cancer diagnosis and prognosis. A complete molecular view of the cancer landscape is necessary for understanding the underlying mechanisms of cancer initiation to improve diagnosis and prognosis, which ultimately will lead to personalized treatment. Interestingly, cancer proteome analysis has also allowed us to identify biomarkers to monitor drug and radiation resistance in patients undergoing cancer treatment. Further, TCGA-funded studies have allowed for the genomic and transcriptomic characterization of targeted cancers, this analysis aiding the development of targeted therapies for highly lethal malignancy. High-throughput technologies, such as complete proteome, epigenome, protein–protein interaction, and pharmacogenomics data, are indispensable to glean into the cancer genome and proteome and these approaches have generated multidimensional universal studies of genes and proteins (OMICS) data which has the potential to facilitate precision medicine. However, due to slow progress in computational technologies, the translation of big omics data into their clinical aspects have been slow. In this review, attempts have been made to describe the role of high-throughput genomic and proteomic technologies in identifying a panel of biomarkers which could be used for the early diagnosis and prognosis of cancer.

## 1. Background

The heterogeneous nature of cancer has tremendously stalled progress in understanding the underpinnings of cancer signaling and its phenotypic manifestation. However, genetic and genomic studies have allowed us to understand the polygenetic nature of cancer, but its effect at the proteomic level is not fully understood. Genomic alterations, such as somatic mutation, have been extensively characterized at the genomic level; however, their phenotypic impact at the proteomic level has not been precisely characterized. Thus, proteogenomic technology is the comprehensive molecular and integrative profiling of genomic alterations, and its impact at the proteomic level has the ability to allow us to decipher precisely the clinical consequences of a mutation. However, current advances in high-throughput proteogenomic technologies have the ability to allow us to enquire into a large number of gene products under a specific experimental condition, which could allow us to precisely unravel altered signaling pathways during tumorigenesis. The comprehensive proteomic analysis of clinical cancer types has tremendously helped us to understand the correlation between copy number alterations and proteome changes [1,2]. The high-throughput proteomic analysis of human tissue samples has shown us the expression of a tissue-specific proteome and long noncoding RNAs (lncRNAs), which play an important role in tumorigenesis and aggression [3,4,5]. The proteogenomic profiling of biomarker signatures in cancer biopsies could be used to monitor response to therapy and at the same time disease progression, as the outcome of a particular treatment is not uniform among affected patients due to specific genomic and epigenomic alterations [6,7]. Thus, based on changes at the individual proteome level, approaches are urgently required that enhance our capability for an early diagnosis of cancer. Thus, a recent upsurge of interest in genomic- and proteomic-based diagnostic and prognostic tools is beginning to usher in a better understanding of the molecular basis of tumorigenesis and the best therapeutic options [8,9].

However, genomic-based alterations are difficult to correlate with a particular disease feature, and it has proven difficult to specify which proteins interact. Recent investigations on human colon cancer have shown that there is a poor correlation between expressed proteome and protein abundance and DNA mutation in the colon cancer genome [8,10]. These studies have shown that tumor-associated somatic mutations or copy number variations might be accountable for these outcomes. The sequencing of individual cancer genomes has underscored the remarkable complexity and heterogeneity in the same cancer subtypes and histo-pathological phenotypes [11]. However, the sequencing of cancer genomes under The Cancer Genome Atlas (TCGA) program has led to tremendous growth in the use of genomic- and proteomic-based technologies for the identification of biomarkers for early cancer detection, molecular targeted therapies, and disease monitoring in various types of cancers [12,13]. High-throughput screening techniques are now being developed to rapidly screen new genes, fused genes, deletion, mRNA transcripts, and proteins. A differential quantitative analysis of these molecules between normal and malignant tissues allows for the identification of gene products and pathways that are dysregulated in a variety of human cancers, including pancreatic cancer [14,15,16]. The application of proteomic technologies has offered a precise mapping of genomic and proteomic alterations and the differential expression of key signaling proteins involved in tumor activation and progression [15]. Oncoproteo-genomics technologies dealing with cancer diagnosis and therapeutics have immense promise to transform clinical practice, including cancer diagnosis and prognosis, and could serve as an alternative to histopathology and the personalized selection of therapeutic combinations that could target a cancer-specific protein network [17,18]. Employing this approach, therapeutic efficacy, toxicity, and drug response could also be monitored in cancer patients, and it delineates deeper insight into neoplasia.

It is estimated that there are 22,000 genes in the human genome, and the properties of the human genome are unvaryingly static [19]. On the other hand, the existence of multiple isoforms of the same gene, post-translational modifications of proteins, and protein–protein interactions provides human proteome with a unique dynamic property [20,21]. Some important studies on cancer proteome have shown that, during tumorigenesis, oncogenic proteins are aberrantly post-translationally modified, and might constitute attractive targets for drugs discovery [22,23]. Interestingly, proteomics has played a lead role in mapping cancer signaling pathways and has successfully delineated the break point cluster region (BCR)-ABL1, extracellular signal-regulated kinase (ERK) pathways, and mechanistic target of rapamycin (mTOR) pathways [23]. Thus, current proteomic technologies are allowing us to analyze individual variations in cancer proteomes and identify the pathways involved in drug resistance, which could be immensely helpful in personalized medicine [8]. Thus, by employing genomic and proteomic tools in a specific cancer subtype, promising biomarkers could be successfully identified as a specific signature pattern associated with it, which could be used either for the early diagnosis or recurrence of cancer [24].

## 2. The Cancer Genome to Proteome

Genomic alterations due to somatic and inherited mutations, copy number variations, and epigenetic changes in cancer genome alter cellular function at the protein level by modulating its abundance and protein–protein interaction [25,26]. The alterations in function are affected differently based on their location and tissue types. Investigations based on cell-line models and patients’ samples have demonstrated that there is no concurrence between copy numbers, RNA level, and protein level [27,28]. Further, the clinical data have shown that the accumulated aberrations at the genomic and transcriptomic levels do not fully represent the structural variations at the proteome level, including post-translational modifications [12]. Interestingly, mutations due to an alteration in gene sequence have a major impact on the structure and function of proteins [12]. Furthermore, a complimentary effort using both the genomic and proteomic-based approaches can be employed to accurately identify genetic variants and their role in tumorigenesis at the protein level [12]. The clinical investigations have shown that the genetic variants of the same gene in different patients have different clinical roles and may have a different clinical outcome [29].

Thus, the proteogenomic analysis of clinical samples may better delineate the functional consequences of somatic mutations and may accurately map driver mutations in significantly larger deletions and amplified regions in chromosomes. In addition, this approach also has promise to identify therapeutic targets [11,30]. An in-depth characterization of tumors employing genomic and proteomic technologies could allow us to glean insight into tumor subsets and identify specific aberrations that could be clinically relevant as biomarkers and therapeutic targets. Furthermore, the characterization of these tumor subtypes may help to identify important diagnostic and therapeutic targets that are specific to a particular subtype. Thus, the genomic- and proteomic-based two pronged approach has the capability to refine present proteomic analysis methods to minimize false positives in identifying targets [31]. Interestingly, the complimentary approach of using both proteomics and genomics has been extremely successful in unraveling aberrantly regulated pathways in ovarian cancer, which might be involved in tumorigenesis [1]. Further, an understanding of how somatic mutations in a cancer patient’s genome alter the pathways in proteome is crucial, which as a result present different clinical phenotypes and therapeutic targets [1,25]. Consequently, an understanding of comprehensive genomic and proteomic alterations would immensely facilitate the identification of alterations in protein–protein interactions and protein kinases phosphorylation/dephosphorylation functional switches at the proteome level, which are responsible for modifying cellular phenotypes.

## 3. Clinical Proteomics in Cancer Diagnosis and Prognosis

The World Health Organization has proposed that the early detection of cancer and proper treatment could save millions of cancer patients worldwide [32]. Early diagnosis and better therapeutic options are desperately needed to improve the survival rate of cancer patients. In most of the cancers, somatic mutations in multiple genes aberrantly trigger tumorigenesis, which is a multistep process, involving a series of specific genetic mutations in each step [33]. Recently, a comprehensive genetic analysis of multiple cancer genomes in pancreatic cancer has shown 63 genetic alterations, the majority of which are point mutations [34]. Interestingly, these alterations have been shown to affect 12 signaling pathways leading to pancreatic tumorigenesis [35]. For example, the current biomarkers for pancreatic ductal adenocarcinoma (PDAC) are not specific and sensitive [36], and as a consequence, high numbers of PDAC cases are diagnosed at advanced stages of disease progression. Even among the 10–20% of PDAC cases where surgical resection is an option, most patients eventually die because of recurrence [37]. The main reason for the failure of current conventional therapy to cure cancers, and the major cause for cancer-related mortality in general, is the ability of malignant cells to detach from the primary tumor site and metastasize in different regions of the same organ and in distant organs [38]. The completed cancer genome projects and sequencing analysis have facilitated the analysis of whole-cancer proteome across tumor types, which has resulted in a better understanding of the tumor landscape [25]. Recent clinical investigations have shown that when the unique set of prostate tumor genomic biomarkers (232 single nucleotide polymorphisms) was combined with six plasma protein biomarkers there was an improved and correct diagnosis of prostate cancer in five clinical subjects as compared with prostate specific antigen (PSA) alone for the detection of tumor with a seven Gleason score [39,40].

Most recently, triple-negative breast cancer proteome data complimented with cancer genome data have revealed that genomic aberrations severely affect protein expression [41]. Further, the combined approach successfully identified markers for drug sensitivity and a pathway for drug resistance [41] in triple-negative breast cancer cells. Interestingly, the combined approach seems to have promise for identifying drug targets and could be immensely useful for personalized medicine [11,42]. Recently, TCGA-sequencing efforts have shown that functional proteome positively complements genomic and transcriptomic data, and this approach has the ability to identify new cancer biomarkers and the underlying biological mechanism [43]. In another pan-cancer study, researchers found discordance between HER2 copy number variation, mRNA expression, and protein expression level in colorectal and serous endometrial cancer [44], which demonstrates that a simple protein-based analysis of patients’ samples across tumor subtypes could highlight potential therapeutic targets. The detailed molecular landscape obtained by this approach could not be concluded by just analyzing either DNA or RNA alone [25,45].

Further, in astoundingly clinical genomics studies of high-grade serous ovarian carcinomas (HGSCs), it has been observed that in more than 96% of ovarian cancer patients TP53 is somatically mutated, which derives neoplasm in ovarian tumorigenesis [46,47]. In addition, HGSC is the most common subtype of ovarian cancer associated with breast cancer susceptibility gene (BRCA) germline mutation in patients. Thus, the genetic testing of patients who have shown a strong family history of ovarian cancer and breast cancer will be immensely benefitted by monitoring TP53 along with copy number variations and DNA methylation as a biomarker [47,48,49]. Further, genomic studies of epithelial ovarian cancer have been able to identify the FGFR4 pathway and its inhibitor BIBF-1120, which has the potential to block cancer progression. These novel tools specify FGFR4 as a potential pathway, which could offer targeted therapy to epithelial ovarian cancer patients with a specific, enriched activation of this pathway [50].

A clinical proteogenomic analysis of 100 pancreatic ductal adenocarcinoma samples was able to precisely identify mutations in TP53, KRAS, SMAD4, CDKN2A, ARID1A, and ROBO2, including mutations in the KDM6A and PREX2 driver genes, which drive PDAC tumorigenesis. In a majority of these samples, a specific group of patients exhibited susceptibility to platinum-based chemotherapy [51]. In a similar study, a genomic analysis of 456 PDAC samples identified 32 genes which impacted or altered 10 signaling pathways, and a further expression analysis of these samples identified four cancer subtypes which could help in designing an appropriate therapeutic options [52]. Thus, by employing genomic, transcriptomic, and proteomic technologies in a clinical setting, the specific signature of biomarkers could be identified for the early diagnosis of pancreatic cancer cases [53]. The proteogenomic-based approach is proving to be immensely important in characterizing breast cancer samples. A proteogenomic analysis of 77 breast cancer samples has shown that 43% of PIK3CA is mutated in luminal breast cancer tumors, and 83% of TP53 is mutated in basal-like tumors (TCGA, 2012). Further, a phosphopeptide analysis of both PIK3CA and TP53 showed that 62 phosphopeptides were upregulated in PIK3CA-mutated tumors, and 56 phosphopeptides were upregulated in TP53-mutated samples [12]. Thus, both PIK3CA’s and TP53’s phosphoporoteome signature could be used as a functional phenotype of breast cancer in patients, and this could also be used to identify therapeutic targets [12,54].

In colorectal cancer (CRC), approximately 65–70% of cases show a genomic instability pattern and mainly the APC, P53, KRAS, and SMAD genes are mutated in a particular subclass of CRC. It has been observed that the copy number variation (CNV) of these genes has been found in a higher percentage of CRC patients, which always alters the gene expression pattern in those patients and has promiscuous impact in treatment response [55]. Interestingly, the CNV of APC, KRAS, TP53, and SMAD has the potential to be used as a biomarker for response to chemotherapeutic drugs such as paclitaxel [56]. A recent investigation has shown that 30–50% of CRC patients encounter recurrence after successful treatment therapy, and currently the carcinoembryonic antigen (CEA) blood test is the gold standard to monitor its recurrence, which lacks sensitivity and specificity to precisely prognose the disease [57]. Based on recent clinical genomics studies, the methylation assay of the BCAT1 and IKZF1 genes has been found to be more sensitive for monitoring CRC recurrence in patients [57].

Thus, it could be envisaged that the use of a panel of biomarkers which shows a specific pattern of somatic mutations at the proteome level and which exhibit differential expression are promising biomarkers in clinical studies.

## 4. Post-Translational Modification of Proteome as Diagnostic and Drug Target

Interestingly, more than 200 different post-translational modifications are identified in proteins. The post-translational modification (PTM) of the proteome is a dynamic process which regulates the cell signaling process. The inherent characteristics of each PTM are capable of generating a functional change in macromolecules that potentially affects cancer activation, progression, and therapeutic response [8].

**Figure 1:**
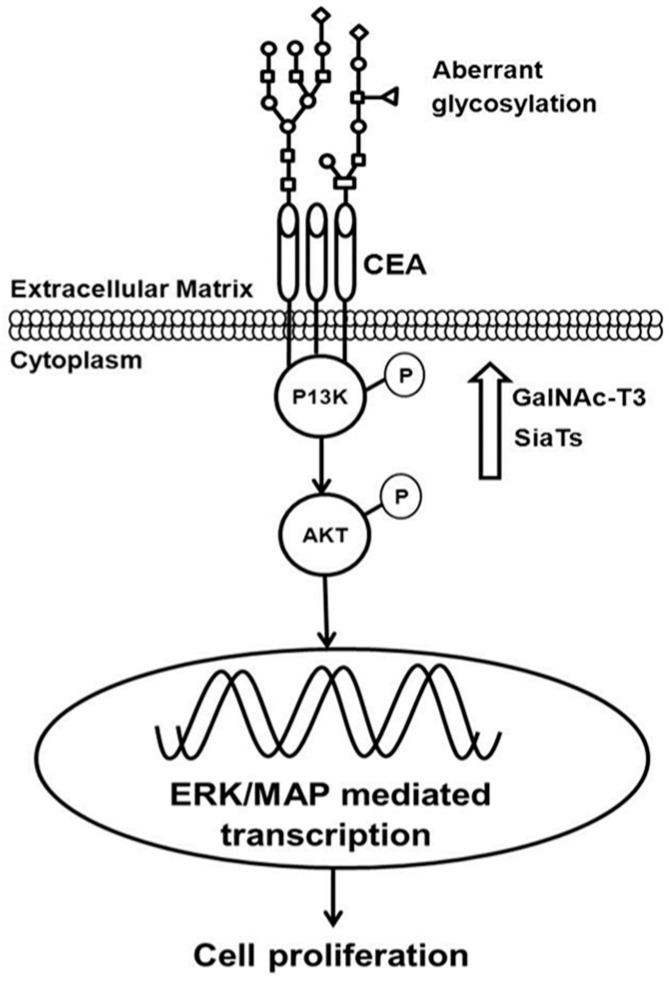
Post Translational Modifications, such as phosphorylation and glycosylation of carcinoembryonic antigen (CEA), phosphoinositide 3-kinase (PI3K) and protein kinase B (AKT), aberrantly activate signaling during tumorigenesis [15] (permission obtained for reproduction).

However, their exact role during tumorigenesis is not well-understood, and cutting-edge mass spectrometry techniques are being used to characterize these modifications. The most prominent modification is phosphorylation, which is aberrantly activated during tumorigenesis (Figure 1) [58,59]. It is a well-established fact that cancer is triggered due to mutations and epigenetic alterations in the genome; however, the molecular impact of these changes transpire at the proteome level by dysregulating signaling pathways [60]. Moreover, quantitative modulation in aberrantly regulated proteins, such as a number of receptor tyrosine kinases and the G Protein-Coupled Receptor, and their differential expression and post-translational modifications enact dysregulation of cell signaling and trigger tumorigenesis [9]. Some post-translational modifications, such as phosphorylation, acetylation, and glycosylation, illicitly activate signaling pathways, which affects normal cellular function [61,62]. Nonetheless, the study of PTMs and their role in cancer diagnosis and prognosis has been very limited [62]. The most investigated modification in cancer research has been STY-based phosphorylation, and its role in cancer research has been significantly characterized [63,64]. Further, the role of glycosylation, ubiquitination, and acetylation in tumorigenesis is also being intensively investigated [65,66].

During tumorigenesis, genetic alterations in signaling molecules lead to over-activated cell surface receptors, which as a consequence affect downstream signaling pathways. Normally, membrane receptors, such as HER2 and FGFR, and also components of the intracellular signaling cascade, such as the K-RAS and ERK kinases, which are quite conserved members of the cell signaling pathway, also join and amplify abnormal signals downstream [67,68,69]. Interestingly, post-translational modification, such as the phosphorylation of STY residues in these kinases, also transiently controls and propagates abnormal signals during tumorigenesis, and as a consequence a cell faces altered signaling pathways [70,71,72]. Recent investigations have shown that genetic alterations, such as somatic or germline mutations, modulate the functional activity of protein kinases, including many receptor tyrosine kinases and phosphatases in the genome, which has a functional impact at the proteome level. In conclusion, genomic alterations due to mutations functionally over-activate or alter signaling pathways, which make cells susceptible to neoplastic growth [73].

Recent investigations have suggested that enhanced STY phosphorylation of onco-proteins in ovarian cancer tissues is associated with a poor clinical outcome [74]; specifically, platelet-derived growth factor receptors in the intracellular tyrosine kinase pathway are activated in some cohorts of ovarian cancer patients with short survival [75]. The TCGA data set analysis of 3185 genomes, which covered 12 tumor types, showed phosphorylated Single Nucleotide Polymorphism variants in 90% of tumors, which could influence cancer susceptibility by modifying the phosphorylation kinase network. Further, 29% of the somatic mutations abolished the phosphorylation and modified kinase target site, which resulted in altered signaling pathways [76]. A recent study in non-small cell lung carcinoma cancer patients has shown a fourfold upregulation of phosphorylation of Akt in lung tumor samples [77], In addition, analogous studies have also suggested that the phosphorylated form of Akt has been associated with a poor prognosis and tumor aggression [78,79,80]. Interestingly, differential phosphorylation was observed in metastatic primary breast cancer tissues in histone H1 at threonine 146. The data have shown a variable phospho staining pattern across different tumor phases and subtypes, which was well-correlated with tumor grades. Thus, H1 phosphorylation at T146 could serve as a clinical biomarker for breast cancer phases [81]. In another elegant study, a specific phosphorylation signature has been linked to a specific lung cancer subtype, which could immensely help in the precise diagnosis and treatment of lung cancer [82].

Human proteome contain 518 kinases, and several of them are phosphorylated during tumorigenesis and determine different forms of phenotypes [83]. A considerable number of these kinases have been identified as promising drug targets in cancer. Phosphorylation has been used as a drug target in cancer since 1998, when Imatinib, a tyrosine kinase inhibitor, was used for treating chronic myelogenous leukemia (CML). Currently, a combination of small molecule kinase inhibitors are being used to treat many cancers, which also delay the onset of resistance [83,84]. The aberrant expression of HSP27 and its enhanced phosphorylation has been observed in breast cancer [85], and recently anti-hsp27 phosphorylation inhibitors are being developed to treat breast and prostate cancers. A recent study on the role of Ybx1 phosphorylation in colon cancer has shown that S176 phosphorylation is responsible for an aggressive form of colon cancer, and its inhibition could be an important treatment option for colon cancer [86].

Glycosylation is another important PTM involved in neoplastic transformation. Aberrant alterations in glycosylation patterns have been linked with tumor aggression and tumor microenvironment heterogeneity [87]. N-linked glycosylation is involved in a variation of cellular functions, such as cell–cell interaction such as metastasis and cancer progression [88]. The discovery of an N-linked glycosylation site is important in the regulation and function of BRCA1 in breast cancer, which is a potential breast cancer biomarker [88,89]. In ovarian cancer, many membrane proteins have been found to be aberrantly glycosylated and modified, including CA125 and KLK6, which qualify as potential biomarkers for an early diagnosis [90]. Further, MUC-4—a transmembrane protein—expression has been observed in pancreatic ductal adenocarcinoma, and it is aberrantly glycosylated, which is involved in cancer progression and neoplast cancer aggression [91]. Thus, MUC-4 can be a useful target in the development of novel therapeutic strategies for the treatment of pancreatic cancer [92]. Additionally, a comparative proteomic analysis of three breast cancer cell lines (MCF-7, MDA-MB-453, and MDA-MB-468) has identified three N-linked glycosylated membrane proteins, namely galectin-3 binding protein, lysosome associated membrane glycoprotein 1, and oxygen-regulated protein, respectively. Analyzing N-glycoproteins from the membranes of breast cancer cell lines highlights potential biomarkers for breast cancer diagnosis and promising therapy [89,93]. Furthermore, genetic and epigenetic modifications on many glycogenes are associated with malignant transformation. Through the recent advancement in proteomic technologies for cancer-cell glycomics, many tumor-associated glycoproteins and glycoproteomics have been exploited for diagnostic, prognostic, and therapeutic purposes [87,94]. Furthermore, tumor-associated glyco-antigens illicitly generate serum antibodies, which have potential applications as biomarkers for early breast cancer detection [95]. The detection of aberrant glycosylated MUC1-specific autoantibodies correlates with colorectal cancer, which has the capability to predict cancer with 95% specificity [96]. However, the low sensitivity of this marker could be used in combination with other markers, suggesting that a combination of antibody signatures may eventually enable a biomarker panel for the early detection of cancer [96].

Acetylation also plays an important role in the regulation of numerous onco-proteins involved in tumorigenesis and cancer progression [97,98]. Protein acetylation is involved in several processes, including cancer [99,100]. Lysine N-acetylation precisely regulates the function of histone and non-histone proteins, and, especially, histone acetyltransferase (HAT) are dysregulated as a result of numerous genetic or epigenetic alterations. Normally, HAT act as tumor suppressors and help cells maintain normal growth and cell cycle and keep control of oncogenes. However, abnormal acetylation could activate malignant proteins and trigger tumorigenesis [101]. Recently, an abnormal acetylation profile has been used as diagnostic marker for early cancer detection. Moreover, acetylation also has potential as a prognostic biomarker to monitor cancer treatment. Further, epigenetic therapy, employing histone deacetylase inhibitors and acetylation modulators, shows promise in treating some forms of cancers [102,103,104]. Some of the proteins involved in controlling N-acetylation and their targets are aberrantly regulated during tumorigenesis, and small molecule inhibitors such KAT, KDAC, and bromodomain are being tested as potential anti-cancer therapies to treat relapsed or refractory cutaneous T-cell lymphoma [105].

In conclusion, post-translational modifications play an extremely important role in cancer activation by altering signaling pathways controlled by kinases. Thus, the phosphorylation of STY influences the kinases-phosphorylation network, which also alters responses to adjuvant therapy. The glycosylation of membrane receptors such RTK and GPCR could also play an important role as a biomarker for disease diagnosis and in monitoring the effectiveness of neoadjuvant and adjuvant therapy.

## 5. Role of Individual and Panel of Biomarker Signatures in Cancer Diagnosis and Prognosis

A few selective OMICS investigations have reported a number of diagnostic biomarkers which have the potential to monitor cancer patient response to chemotherapy and radiotherapy [106,107,108]. Currently, there is a dearth of potential genomic or proteomic biomarkers for monitoring radiation therapeutic response and its success in a cancer patients, and this poses serious challenges in oncology [9]. Nonetheless, a very few prognostic assays have been optimized, but none of these markers have been proven to be promising in a clinical setting. Thus, advances in the proteogenomics approach have immensely helped the field of cancer research to successfully discover new predictive biomarkers which could monitor the effectiveness of cancer therapy. Further, progress in high-throughput genomic and proteomic technologies has helped us to identify new specific and sensitive biomarkers which have clinical significance [109,110]. Earlier studies have reported the use of a single biomarker which has shown significance in the early diagnosis of cancer, and by employing a proteogenomic approach, the APEX1 gene has been identified as biomarker which could monitor damaged DNA repair, and its deletion triggered radiosensitivity in cell lines inherently expressing radio resistance phenotypes [111]. Recently, a Genome Wide Association Studies analysis in prostate cancer samples has shown a TANC1 locus linked to radiation-induced toxicity [112]. Additionally, a number of gene variations have also been associated with radiotoxicity. Furthermore, cathepsin D and peroxiredoxin-5 have been found to be upregulated in breast cancer cell lines in response to radiation therapy [113]. In a corollary finding, CXCR4 has been well-characterized as a marker to monitor radiation resistance in cancer stem cells [114].

The earlier researches have been confined to single biomarkers, and a number of reports have characterized a single biomarker for the prediction of early cancer diagnosis and prognosis [111,115]. However, it has been observed that single proteomic biomarkers lack the precision to accurately detect cancer and at the same time its clinical effectiveness is very limited [8,111,113,116]. Consequently, the use of multigene expression signatures or a panel of proteomic signatures in a tumor sample, showing a distinct expression pattern and displaying an enhanced diagnostic precision are promising candidates as early stage biomarkers [106]. Interestingly, a set of multi-gene signatures has been employed as a biomarker to prognosticate radiation therapy and sensitization to therapy in prostate cancer treatment [117,118]. In another study, a set of gene and protein signatures has been used to monitor radiation resistance response in head-and-neck and breast cancer samples [119,120]. Thus, based on these studies, it is envisaged that specific genomic and proteomic signatures seem to be specific and effective prognostic markers in breast, lung, and head-and-neck (HNC) cancers [121,122]. In a similar approach, distinctive genomic signatures have also been employed to monitor cancer therapy and drug response in some cancer patients. Consistently, an amalgamation of protein signatures are more authentic and unfailingly predict tumor response to radiation therapy and normal tissue response, which seems to be essentially important for monitoring clinical response [110,123,124]. In another elegant study based on Reverse Phase Protein Array (RPPA) analysis of a cohort of 118 stage II colon cancer patients, an upregulation of three components of an activated PIK3/Akt pathway, namely phospho-Akt, S6RP, and phospho-4E-BP1, served as novel biomarkers for stage II colon cancer recurrence [125]. Thus, an integrated genomic and proteomic profiling of cancer samples presents a comprehensive analysis which helps in the discovery of pathways involved in drug sensitivity and drug resistance [126].

## 6. The Role of Genomics and Proteomics in Personalized Cancer Care

After the completion of human genome, a large number of genes have been mapped and their precise role in disease progression and the role of the environment have also been deciphered [127,128]. Thus, genomics have played an immensely important role in understanding the disease process more precisely and have led to better targeted therapies. The genomic mutational landscape of a cancer patient is immensely helpful to precisely diagnose and design treatment options. A large number of cancer genome sequence analyses have shown that each patient’s tumor contains specific genetic alterations and that these alterations drive tumorigenesis [129]. Interestingly, a clear landscape of genomic alterations in a specific cancer subtype may lead to an individualized medical treatment that is precisely based on the specific genetic alterations of an individual cancer patient [11]. Genetic and genomics alterations in individual cancer types and subtypes could play an immensely important part in advancing precision medicine through Next-Gen sequencing and bioinformatics analyses. The comprehensive genomic profiling of a tumor sample could help in tracking key genomic changes, and this could immensely help in designing and developing more effective options to precisely diagnose and effectively treat cancer patients [130].

Investigations on numerous and diverse cancer genomes have shown that cancer pathologies are not restricted to a single genomic event. A large-scale cancer genome atlas (TCGA) analysis has clearly established that a large number of genetic alterations and aberrations in an individual genome and epigenome accumulate over time and activate malignant transformations [131]. These alterations are quite unique to a specific tumor, and are being exploited for specific diagnosis and treatment [132,133]. This approach has enabled the identification of specific biomarkers which are now being used for diagnosis, prognosis, and therapeutic decisions in cancer patients. The well-established examples of a precision medicine approach are the mutational profile of the EGFR receptor gene, which triggers uncontrolled growth and at the same time blocks apoptotic signals. Thus, Tarceva is designed to block mutant EGFR activity [134,135]. In a corollary finding, Cetuximab therapy in colorectal cancer patients based on a KRAS mutational profile has been quite promising [135].

**Figure 2:**
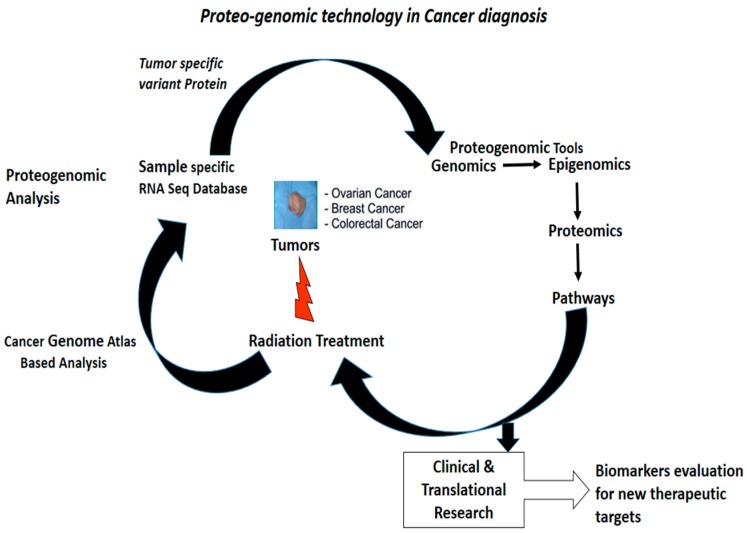
Proteogenomic steps to identify and characterize biomarkers which are therapeutically significant for precision medicine [8]. (Permission obtained for production)

The cancer genome sequence analysis and our expanding knowledge of cancer mutation landscapes have paved the way for personalized medicine. Genomics extends a clinical practitioner’s access to multiple genetic tests that allow them to determine which genetic variants exist in their patients. Genetic tests, such as genotyping, Comparative Genomic Hybridisation (CGH) arrays, exome and whole genome sequencing, may provide a clear understanding about the specific genetic variants present in a biopsy sample [8,136]. Interestingly, the genetic profiling of tumor samples helps us to understand the link between specific genetic variants and the important clinical state of the patient, which could serve as an important step in personalized medicine (Figure 2) [135,136]. Further, patient-specific genetic information combined with other clinical data could pave the way for best personalized treatment. Thus, because of the early success of the ABL1 kinase inhibitor Imatinib to target the BCR–ABL1 fusion protein in Chronic Myeloid Leukemia, genomics-based clinical characterization has now become the standard of care for some cancers [137]. For example, in lung adenocarcinoma, EGFR testing for mutations and ALK rearrangements allows for precision therapy with targeted kinase inhibitors, such as gefitinib for EGFR and Crizotinib for ALK fusion [137].

## 7. The Cost Effectiveness of Precision Medicine in a Health Care System

Due to advancement in genomic and proteomic technologies, personalized medicine is emerging as a popular choice of medical treatment in health care systems. Interestingly, genomic and proteomic biomarkers are being used to determine the appropriate treatment options for each patient, and health experts are using the best information management tools to access and share patient pathological data to develop targeted therapies. However, there has been slow progress in immaculately calculating the cost of precision medicine [138]. Further, due to advances in proteogenomic and pharmacogenomics technologies, personalized cancer care has immense opportunity to lower the cost of cancer treatment [138,139]. Interestingly, the novelty for personalized medicine is the prospect of introducing a new personalized health care model in patient care. The cohort of patients who respond to a particular treatment based on their genomic, proteomic, and pharmacogenomic profiling while avoiding side effects not only can change the dynamics of personalized medicine but also the practice of medicine [139,140]. Since the completion of the human genome, there has been slow progress in the development of precision medicine in spite of investing 1 billion dollars U.S. and 13 years in mapping the human genome. During that time, sequencing technology evolved from the manual Sanger sequencing method using radioactive labels to automated sequencing using color-coded fluorescent dyes. Genome sequencing costs came down from $100 million in 2001 to about $10 million in 2007. During 2011, the cost and duration of sequencing an entire genome had decreased to $50,000; quickly thereafter, Illumina announced that it had lowered the price for sequencing whole human genomes to $5000 per genome, and by 2014, Illumina sequenced a human genome for only $1000. It is also envisaged that additional costs and time are necessary for genome analysis and annotation in a clinical setting. As the cost and duration of genomic sequencing continues on a sharp downward curve, many medical experts believe that the cost of one genome sequence will come down to $700 or less, and this will be considered a benchmark because it is comparable to costs of present medical tests and procedures, and could enable them to begin to accept patients for personal genome analysis, while full genome analysis services are already in practice to resolve difficult diagnoses, with insurers determining that the approach was cost-effective enough to be reimbursed for personal care [141]. Thus, practicing personalized medicine could facilitate early diagnosis and help design targeted treatments which will result in better clinical outcomes.

## 8. Future Perspective

Cancer genomic alterations, combined with cutting-edge proteomic technologies and bioinformatics tools, are beginning to reveal patients’ specific cancer landscapes. Thus, an onco-proteogenomic approach can precisely identify point mutations, splice variants, copy number variation, and gene fusions in a patient’s genome, which could be complimented with changes in the proteome and its post-translational modifications, which could immensely facilitate the early diagnosis, prognosis, and treatment of cancer. Further, the continuous downward slide in genome sequencing and analysis cost is paving the way for more cost-effective precision medicine. Recently, metabolomics is becoming a powerful tool to investigate cancer progression, and it has been found that during tumorigenesis new metabolites are synthesized due to altered signaling pathways. Thus, by employing metabolomics tools, alterations in metabolite expression could be profiled and at the same time their quantification could be measured in a patient’s sample. Furthermore, by metabolic profiling, biomarker signatures could be identified which might be immensely helpful in the diagnosis of cancer and monitoring therapeutic response of a particular treatment, including cancer recurrence and resistance to drugs.

Advances in Next-Gen sequencing have facilitated the RNA-Seq-based transcriptomic analysis of cancer samples, and it allows us to precisely quantify mRNA transcripts and helps us to identify the various genetic variants which might be responsible for cancer progression. An RNA-seq-based transcriptomic analysis can precisely identify those genetic variants which contain single nucleotide somatic mutation and splice forms. Thus, using this approach, multi-gene mRNA expression signatures could be identified in specific cancer subtypes in breast cancer and an appropriate treatment decision could be made. Thus, a precision medicine approach could reveal the clinical phenotype of the tumor and could be used to monitor therapy success, drug, and radiation resistance.
